# A Masterwork of Art, a Metaphor for Prevention

**DOI:** 10.3201/eid2407.AC2407

**Published:** 2018-07

**Authors:** Byron Breedlove, Anne Schuchat

**Affiliations:** Centers for Disease Control and Prevention, Atlanta, Georgia, USA

**Keywords:** art science connection, emerging infectious diseases, art and medicine, about the cover, Pieter Bruegel the Elder, The Wine of Saint Martin’s Day, A Masterwork of Art, a Metaphor for Prevention, vaccine-preventable diseases, public health

**Figure:**
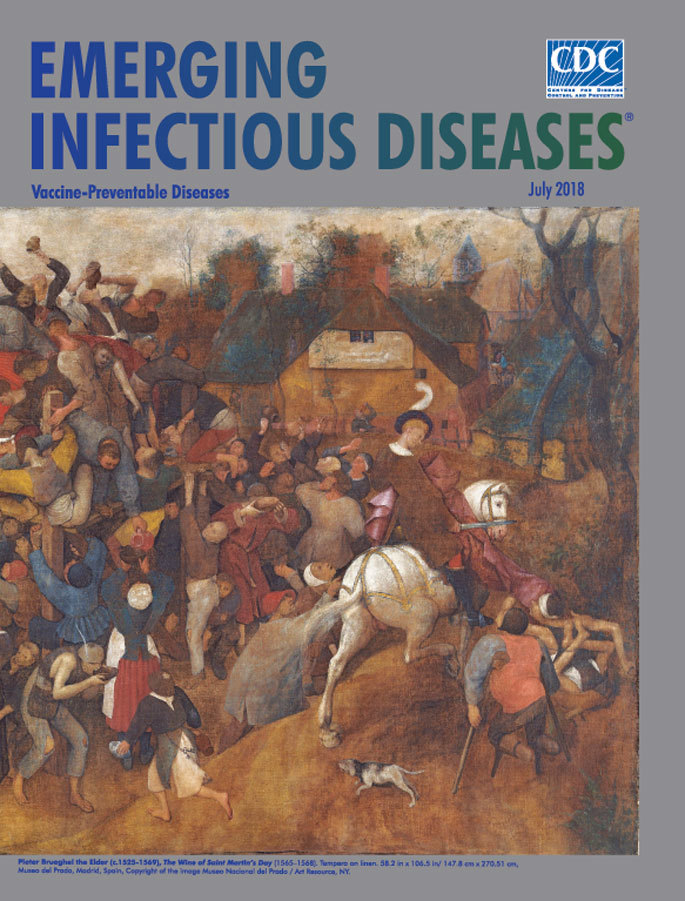
**Pieter Bruegel the Elder (c.1525–1569), *The Wine of Saint Martin’s Day* (1565–1568).** Tempera on linen. 58.2 in × 106.5 in/147.8 cm × 270.51 cm, Museo del Prado, Madrid, Spain, Copyright of the image Museo Nacional del Prado/Art Resource, NY.

Pieter Bruegel the Elder, the best known member of a large family of artists who were active in the 16th and 17th century Netherlands, is acclaimed as one of the most important 16th century Dutch and Flemish Renaissance painters. Art critic Johnathan Jones notes that “The genius of Bruegel shows itself not just in his wild imagination—in which he resembles the southern Dutch visionary Hieronymus Bosch—but his acute feeling for landscape and human behaviour.”

*The Wine of Saint Martin’s Day* is the largest of the 41 surviving paintings from this Flemish master and the last to be included among his oeuvre. In 2010, staff from the Museo del Prado verified that this painting was an authentic Bruegel. According to the museum’s documents, this work is a *tüchlein*, painted in glue-size tempera on unprimed linen. Although many artists in 15th and 16th century Flanders used this technique, few examples have survived.

Bruegel’s vivid, detailed representations of Flemish village life—the festivals, feasts, celebrations, weddings, and hunts—offer a durable record of a folk culture lost to time. The festival associated with Saint Martin's Day on November 11 was an occasion for feasting and revelry ahead of the oncoming winter. Central to the celebration was the new wine of the year, known as Saint Martin’s wine. Art critic Michael Kimmelman describes Bruegel’s sprawling painting as “a panoramic canvas showing a mountain of revelers drinking the first wine of the season, and a few of them suffering its consequences.” Bruegel’s calculated focus on details permeates his unfolding spectacle of avarice. As Kimmelman notes, “You can admire the delicacy of faces and hands and feet, alive and varied, making a jigsaw of humane detail, Bruegel’s trademark. . . .”

The Museo del Prado describes revelers clustered at the center of the painting: “Around the barrel the artist has arranged a varied crowd of figures: old and young men, women, some with children, peasants, beggars and thieves, all trying to obtain the largest possible quantity of wine. Those who have been successful and have filled their containers with wine are back on the ground while others are still clinging onto the wooden supports, lying on the barrel or leaning over perilously to catch the wine as it spurts from the barrel in whatever recipients they have to hand, including their hats and shoes.”

In addition to the consequences from overindulgence in wine and food, this crowd of revelers, regardless of social status or age, could have shared something more insidious at the festival. Respiratory infections, which are easily spread among people who gather in close proximity, can be transmitted from person to person through respiratory droplets in coughs or sneezes or through contact with fomites contaminated by the respiratory droplets. Late autumn, when the November celebration of St. Martin’s Day occurs, typically heralds the onset of cool, dry weather in temperate zones in the Northern Hemisphere and the season when pneumonia and influenza infections begin to increase.

Bruegel liked to place key actions away from the viewer’s focus, as is evident in the more familiar work *The Fall of Icarus* captured in the W.H. Auden poem *Musée des Beaux Arts*. For *The Wine of Saint Martin’s Day*, if the viewer’s eyes wander to the blue-topped towers dwarfing a collection of small figures, scan the just discernible horizon, alight on the smattering of trees, or glance at the figure astride a white horse placed in the periphery, they invariably return to the 100 or so figures splayed across the canvas and that teeming bolus of activity around the huge red barrel.

The Heilbrunn Timeline of Art History notes that Bruegel’s paintings “. . . demonstrate the artist’s attentive eye for detail and attest to his direct observation of village settings, they are far from simple re-creations of everyday life. The powerful compositions, brilliantly organized and controlled, reflect a sophisticated artistic design.” The Museo del Prado stresses that “The artist creates a deliberate contrast between the central group around the barrel and the much more stable, pyramidal group that depicts the charity of Saint Martin on the right.”

St. Martin’s act of cutting his cape in half to help provide warmth to a poor man is nearly overlooked, taking place at the lower right corner of the canvas. St. Martin’s cape could be a metaphor for the protection from disease afforded by vaccines, the similarity being that prevention rarely garners the spotlight and sometimes may seem overlooked. However, immunization can provide protection against many respiratory and other vaccine-preventable diseases. 

Although many emerging infections derive from exotic wildlife reservoirs or invasive mosquito and tick species, most respiratory and vaccine-preventable infectious diseases represent the mundane, previously common infections that do not generate headlines. Nonetheless, their control and even elimination represent a masterwork of public health.
